# Deficiency of SARM1 attenuates neuronal injury and improves neurological performance in a photothrombotic stroke model

**DOI:** 10.1186/s13041-025-01251-5

**Published:** 2025-11-21

**Authors:** Yanjie Huang, Xiaofeng Cheng, Ke Yan, Yufan Ma, Qingwu Yang, Sen Lin

**Affiliations:** 1 Department of Neurology, Second Affiliated Hospital of Army Medical University, Chongqing, 400037 China; 2 Chongqing Institute for Brain and Intelligence, Guangyang Bay Laboratory, Chongqing, 401339 China

**Keywords:** SARM1, Stroke, Neuron injury, Axonal degeneration, Neuroprotection

## Abstract

Stroke is a major cause of morbidity and mortality worldwide. There is an urgent need for effective neuroprotective agents to reduce brain injury. SARM1 (sterile alpha and TIR motif-containing 1) has been identified as a key mediator of axonal degeneration. However, its role in stroke and the underlying mechanisms remain insufficiently understood. In the present study, a mouse model of stroke with focal infarction in the cortex was used to investigate the potential relation between SARM1 and post-stroke brain injury. We found that SARM1 expression increased in neurons of the peri-infarct cortex at an early stage after photothrombotic stroke induction (PTI) and was evenly distributed between excitatory and inhibitory neurons. Deficiency of SARM1 improved neurological performance, reduced the infarct volume and the inflammatory response including reactive gliosis and TNF-α level after PTI. Meanwhile, SARM1 deficiency promoted neuronal preservation in the peri-infarct cortex and mitigated axonal degeneration, possibly because of reduced NAD^+^ consumption of neurons in the peri-infarct cortex. Additionally, we found that SARM1 deficiency inhibited glial scar formation and decreased activated microglia. FK866 and DSRM-3716, two recently reported pharmacological inhibitors of SARM1, failed to alleviate brain injury in mice with stroke. Our findings demonstrate that SARM1 deficiency attenuates ischemic neuronal injury and improves neurological performance post PTI, suggesting that the SARM1 signaling pathway could serve as a potential therapeutic target for stroke in the future.

**Supplementary Information:**

The online version contains supplementary material available at 10.1186/s13041-025-01251-5.

## Introduction

Stroke is a clinical syndrome characterized by acute neurological deficits resulting from disruption of blood supply to the affected brain tissue. Affecting one in four people worldwide, stroke is the second most common cause of death and third leading cause of chronic disability, placing a serious burden on the global economy [8]. Intravenous recombinant tissue-type plasminogen activator is the only drug therapy currently approved for clinical use [18]. Endovascular thrombectomy is recommended for patients with large vessel occlusion who fail to respond to IV therapy. Unfortunately, this treatment has a strict time window for effectiveness, and its availability is limited to major stroke centers, making it inaccessible in many parts of the world. Furthermore, more than 40% of patients experience disability or death within 90 d despite vascular recanalization [3]. Our recently published randomized clinical trial examined the effects of adjunct intra-arterial urokinase and tenecteplase after near-complete reperfusion with endovascular thrombectomy. Results indicated that adjunct intra-arterial urokinase and tenecteplase did not significantly improve the likelihood of survival without disability at 90 d in patients with acute ischemic stroke due to large vessel occlusion [14, 22]. Therefore, there is an urgent need for effective neuroprotective agents to mitigate brain injury. Currently, several neuroprotective agents, including free radical scavengers, calcium channel blockers, excitatory amino acid modulators, and magnesium ions, have demonstrated potential benefits in preclinical experiments. However, none have proven effective in human clinical trials [27].

In addition to direct injury caused by ischemia, a cascade of secondary events, including oxidative stress, blood–brain barrier damage, brain edema, glial cell activation, inflammatory mediator release, and phagocytosis triggered by leukocyte infiltration, further exacerbates neuronal degeneration [5, 31]. However, no currently available therapy targets active neuronal degeneration in ischemic stroke. It is therefore hypothesized that inhibiting neuronal degeneration could reduce neuronal death and improve outcomes in patients with stroke. SARM1, an nicotinamide adenine dinucleotide (NAD^+^) hydrolase, plays a central role in neuronal degeneration. Under normal conditions, nicotinamide mononucleotide (NMN) adenylyltransferase 2 is transported along axons, converting NMN into NAD^+^. Following injury, NMN adenylyltransferase 2 is depleted from the distal axon, leading to a decline in NAD^+^ and an accumulation of NMN. This imbalance in the NAD^+^/NMN ratio activates SARM1, triggering rapid NAD^+^ degradation, ATP depletion, Ca^2+^ influx, and cell lysis [9]. Thus, SARM1 is a critical mediator of axonal degeneration. Tao Xue et al. found that overexpression of SARM1 harboring a serine-548-alanine mutation reduced infarct volume and improved behavioral performance [35]. However, the exact role of SARM1 in neurons of stroke-affected mice in vivo, as well as its underlying mechanisms, requires further investigation.

In this study, we examined the relation between SARM1 expression and post-stroke neuronal injury. We found that SARM1 deletion promoted neuronal preservation in the peri-infarct cortex and alleviated axonal degeneration, thereby improving behavioral performance after stroke. This effect may be attributed to reduced NAD^+^ consumption in the peri-infarct cortex. Our findings suggest that SARM1 is a promising target for post-stroke neuroprotection.

## Materials and methods

### Animals

Eight-week-old *Sarm1*^⁻/⁻^ mice (body weight, 23–25 g) were obtained from Cyagen Biosciences, Inc. (Jiangsu, China; license no. SYXK (Su) 2020–0006). Eight-week-old male C57BL/6 mice (body weight, 23–25 g) were provided by Army Medical University. All mice were raised in specific pathogen-free animal facilities and maintained at room temperature (23 ± 1 °C) with free access to food and water. All mice were maintained on a standard 12-h light–dark cycle. All animal handling procedures were approved by the Laboratory Animal Welfare and Ethics Committee of Army Medical University (AMUWEC20234728) on February 20, 2023. All experiments involving animals were performed in accordance with the National Institutes of Health Guide for the Care and Use of Laboratory Animals [26].

### Photothrombotic stroke model

A photothrombotic stroke model was used to induce focal infarction in the right cortex. Anesthesia was induced using isoflurane (RWD Life Science, Shenzhen, China; Cat# R510 22–10) in an anesthetic box. The oxygen flow rate was adjusted to 1 L/min, and the isoflurane flow rate was 3 L/min. The isoflurane flow rate was then adjusted to 1 L/min for continuous anesthesia via the nasal cavity during surgery. Anesthetized mice were placed in a stereotaxic frame and intraperitoneally administered 10 mL/kg Rose Bengal solution (10 mg/mL in isotonic sodium chloride solution, Sigma, USA, Cat# 330,000). After 10 min, the skull was exposed through a midline scalp incision, and thinned using a cranial drill until the arterioles of the targeted cortex were clearly visible. The underlying brain tissue was illuminated for 10 min using a fiber optic light guide (cold light source, 2 mm diameter, 34 mW) positioned at anterior–posterior 0 mm and medial–lateral + 1.5 mm relative to the bregma, targeting the border between the primary somatosensory and motor cortices, as shown in Supplementary Fig. 1A. After illumination, the scalp was sutured, and the mice were placed on a heating pad for 1 h to recover from anesthesia before being returned to their home cage. Except for illumination, the sham-operated mice underwent the same surgical procedure [32–34].

### Middle cerebral artery occlusion/reperfusion model (MCAO/R)

Transient focal cerebral ischemia was induced in mice by intraluminal occlusion of the left middle cerebral artery, as described previously [4, 13, 19]. Briefly, the mice were anesthetized with 1% pentobarbital sodium. The left carotid artery was exposed and divided upward to the bifurcation of the internal and external carotid arteries. The external carotid artery was exposed, sutured at the distal end, and transected. A 2-cm length of rounded-tip nylon monofilament (Jialing, Shanghai, China, Cat# L1800) was inserted into the internal carotid artery and advanced to block the left middle cerebral artery until slight resistance was experienced. After 60 min of occlusion, reperfusion was initiated by removing the nylon monofilament. The rectal temperature was maintained within 37.0 ± 0.5 °C during the procedure. The mice were placed on a heating pad before recovering from anesthesia and were then returned to their home cages.

### Microinjection

Adeno-associated viruses (AAVs) were used to anterogradely trace neurons and axons projecting to the spinal cord [25]. Mice were anesthetized with isoflurane (RWD Life Science, Shenzhen, China,Cat# R510 22–10). The oxygen flow rate was adjusted to 1 L/min. The isoflurane flow rate was initially 3 L/min in an anesthetic box and was then adjusted to 1 L/min for continuous anesthesia via the nasal cavity during the injection procedure. The coordinates for AAV injection relative to the bregma were anterior–posterior 0 mm, medial–lateral + 1.5 mm, and dorsal–ventral + 1 mm. rAAV-VGLUT2-EGFP-WPRE-pA (BrainVTA, China, PT-1886) and rAAV-VGAT1-EGFP-WPRE-pA (BrainVTA, China, PT-3176) were diluted in sterile phosphate-buffered saline (PBS) (1:5). ScAAV-hSyn-EGFP-WPRE-pA (BrainVTA, China, PT-2315) was diluted in sterile PBS (1:10). To mark excitatory neurons, 300 nL of rAAV-VGLUT2-EGFP-WPRE-pA was injected into the cortex in the experiment for identifying neuron subtypes. To mark inhibitory neurons, 300 nL of rAAV-VGAT1-EGFP-WPRE-pA was injected into the cortex. One mouse was injected with only one type of rAAV. To label resident pyramidal neurons and trace axons, 100 nL of ScAAV-hSyn-EGFP-WPRE-pA was injected into the cortex in the experiment for observing the morphology of neuronal axons. All injections were administered at a constant rate of 20 nL/min using a microinfusion pump (Hamilton, Switzerland). After infusion, the needle was left in place for 5 min to allow for diffusion and to prevent reflux. The solution was then withdrawn slowly. All coronal Sects. (30-μm thickness) without staining were imaged using an Olympus confocal microscope. Marked neurons and axons were visualized through the expression of green fluorescent protein following AAV infection.

### Neurological deficit score

Neurological dysfunction was evaluated using a 5-point scoring system based on the Longa score test, as previously described [13, 19, 21]. The scoring standards were as follows: zero = no neurological deficit,one = failure to fully extend the contralateral forepaw; two = circling to the contralateral side; three = falling to the contralateral side; and four = absence of spontaneous locomotor activity.

### Infarct volume evaluation

The brains of stroke-induced mice were sliced into an average of seven sections and incubated in a 2% 2,3,5-triphenyl-2H-tetrazolium chloride (TTC) solution (Sangong Biotech, China, Cat# A610558) for 15 min at 37 °C. Normal brain tissue was stained red, whereas infarcted tissue remained white. Infarct volume was analyzed using ImageJ according to the following formula: [(left hemisphere volume − uninfarcted tissue volume of the right hemisphere)/Left hemisphere volume × 100%] [13, 19, 21].

### Immunofluorescence staining

Mice were anesthetized with urethane and perfused with PBS, followed by 4% paraformaldehyde. The brains were extracted and fixed in 4% paraformaldehyde overnight at 4 °C. A 20% or 30% sucrose solution was used for dehydration, after which the brains were embedded in cryomatrix and sectioned into 30-μm slices using a Leica vibratome (CM-1950, Leica, Heidelberg, Germany). Sections were blocked in PBS containing 5% bovine serum albumin (Beyotime, China, Cat# ST023) and 0.2% Triton X-100 (Beyotime, China, Cat# P0096) for 1 h at room temperature. Subsequently, sections were incubated with primary antibodies overnight at 4 °C. The primary antibodies were as follows: SARM1 (1:500, Abcam, Cat# ab17812, RRID: AB_444031), NeuN (1:500, Abcam, Cat# ab279297, RRID: AB_3095692), Iba1 (1:500, Abcam, Cat# ab5076, RRID: AB_2224402), GFAP (1:500, Abcam, Cat# ab53554, RRID: AB_880202), SMI-32 (1:500, Abcam, Cat# ab801701, RRID: AB_2564642) and Nestin (1:500, Abcam, Cat# ab105389, RRID: AB_2251136). The next day, sections were washed three times and incubated with the appropriate fluorescent secondary antibodies for 2 h at 25 °C in the dark. The secondary antibodies (all from Invitrogen) were as follows: Alexa Fluor 555 (1:500, donkey anti-mouse, Cat# A32773), Alexa Fluor 555 (1:500, donkey anti-rabbit, Cat# A31572), Alexa Fluor 488 (1:500, donkey anti-rat, Cat# A21208), and Alexa Fluor 488 (1:500, donkey anti-goat, Cat# A32814). Sections were then incubated with 6-diamidino-2-phenylindole (1:5000, Beyotime, China, Cat# C1002) for 15 min. Finally, the sections were rinsed and mounted onto microscope slides. Confocal images were acquired using an Olympus confocal microscope and processed using ImageJ software.

### Quantitative real-time PCR (qPCR)

Total RNA was extracted from mouse brain tissue using TRIzol reagent (Thermo Fisher Scientific, USA, Cat# 15,596,026). The RNA was then reverse-transcribed into cDNA using the PrimeScript RT kit (Takara Biotechnology, China, Cat# RR047A). Quantitative PCR was performed using the PowerUp™ SYBR Green Master Mix (Cat# A25742, Thermo Fisher Scientific) according to the manufacturer’s instructions. The cycling conditions were as follows: 50 °C for 2 min, 95 °C for 2 min, followed by 40 cycles of 95 °C for 15 s and 60 °C for 1 min. Cycle threshold values were normalized to *β-actin*, and all samples were analyzed in triplicate. Relative quantification was performed using the comparative 2⁻^ΔΔCt^ method. The primer sequences used for quantitative real-time PCR were as follows: *Sarm1*_forward: 5'-ATGACTGCAAGGACTGGGTG-3'; *Sarm1*_reverse: 5'-AAACTGGTATCCGATCCGGC-3.'

### Western blotting (WB)

Mice were anesthetized with isoflurane and perfused with PBS. The brain and spinal cord were carefully extracted under cold conditions. Protein was extracted using RIPA buffer (Beyotime, China, Cat# P0013) supplemented with PMSF (Beyotime, China, Cat# ST505). Protein concentration was determined using the BCA protein assay kit (Beyotime, China, Cat# P0009). Fifty micrograms of protein per lane were loaded onto a sodium dodecyl sulfate–polyacrylamide gel, separated by electrophoresis, and transferred to polyvinylidene fluoride membranes (Millipore, USA, Cat# HATF00010). The membranes were blocked with 5% non-fat milk (Beyotime, China, Cat# P0216) in Tris-buffered saline containing Tween 20 for 1 h. The membranes were then incubated overnight at 4 °C with primary antibodies against β-actin (1:1000, Abcam, Cat# ab6276, RRID: AB_2223210), SARM1 (1:1000, Abcam, Cat# ab17812, RRID: AB_444031), PARP1 (1:1000, Abcam, Cat# ab191217, RRID: AB_303308), and SIRT1 (1:1000, Abcam, Cat# ab189494, RRID: AB_2185378). After incubation, the membranes were washed three times with Tris-buffered saline containing Tween 20 and incubated with HRP-conjugated secondary antibodies (Beyotime, China) for 2 h at room temperature: goat anti-mouse (1:1000, Cat# A0216) or goat anti-rabbit (1:1000, Cat# A0208). After three washes, protein bands were visualized using the BeyoECL Plu**s** kit (Beyotime, China, Cat# P0018S) and quantified using ImageJ software.

### Behavioral test

The grid-walking test was used to assess walking ability and forelimb coordination in mice. A 10 × 10 mm square wire grid was fixed 100 cm above the laboratory bench, allowing mice to walk freely. Each time the contralateral paw slipped through an open grid, a “foot fault” was recorded. Two researchers independently documented the number of contralateral paw slips through the mesh and total number of steps taken on the grid. The experiment lasted 1 min, and data were excluded if a mouse took fewer than 20 steps within this period. Foot fault rates were calculated as foot faults divided by the total number of steps. For the adhesive removal test, two square adhesive tapes (25 mm^2^) were uniformly attached to the forepaws with equal pressure. The time required to remove the tape from each forepaw was recorded. If a mouse required more than 120 s to remove the tape, the time was recorded as 120 s. The test was repeated three times with 10-min intervals between trials. Results were calculated as follows (time in seconds): time for the left paw − time for the right paw. All mice were trained for 3 d before photothrombotic stroke induction [17, 20, 36].

### Enzyme-linked immunosorbent assay (ELISA)

Inflammation-related cytokines including TNF-α and IL-10 were detected and quantified 3 d after PTI. Mouse TNF-α ELISA Kit (JL10484), and Mouse IL-10 ELISA Kit (JL20242) were provided by Jianglai Industrial Limited By Share Ltd., Shanghai, China. All the experimental procedures were performed following the instructions recommended by the manufacturer. The cytokine concentrations were determined based on standard protein concentration.

### NAD^+^ detection

NAD^+^ levels were measured using an NAD/NADH-Glo assay kit (Promega, USA, Cat# G9072) following the manufacturer’s instructions. Briefly, tissues were lysed in base solution (0.2 N NaOH, 50 μL) containing 1% dodecyl trimethylazanium bromide (Sigma-Aldrich, USA, Cat# D8638). Subsequently, 25 μL of 0.4 N HCl was added to the lysed tissue samples (50 μL), and the lysates were heated at 60 °C for 15 min to selectively degrade NADH. The samples were then incubated at room temperature for 10 min. Each acid-treated sample was mixed with 25 μL of 0.5 M Trizma base to neutralize the acid. A total of 100 μL of NAD/NADH-Glo™ detection reagent was added to each well, and the lysates were incubated for 30 min at room temperature. Luminescence was recorded using a luminometer. A 2 mM NAD⁺ solution (Sigma-Aldrich, USA, Cat# N8285) in PBS was used to generate a standard curve, and luminescence (in RLU) was converted to NAD⁺ concentration.

### Sholl-analysis and cell body-to-total cell size ratio

Sholl-analysis was used to analyze microglial morphology [20]. After selecting the field of view around the infarction, Olympus microscope was used to capture pictures, with each image consisting of 20 slices with a 0.5 μm interval. The original data were processed and analyzed using Image J. For Sholl analysis, the total branch length, the number of terminal points were recorded. For the cell body-to-total cell size ratio, soma volume and total cell size were recorded. Three cells with intact dendritic trees were selected from each image, and the average value was ultimately included in the statistics.

### Pharmacological interference with FK866 or DSRM-3716

Mice with photothrombotic stroke were treated with FK866 (10 mg/kg, intraperitoneally, Cat# F8557, Sigma-Aldrich) twice daily until the experiment was terminated [23]. For DSRM-3716 (Cat# HY-W021879, MedChemExpress, USA), mice received a daily intraperitoneal injection of 10 mg/kg for 3 d post PTI. Control mice were injected intraperitoneally with an equivalent volume of solvent.

### Statistical analysis

Data were presented as means ± SEM. Statistical significance was assessed using Student’s *t*-test for comparisons between two groups. One-way ANOVA was used for multiple-group comparisons. Behavioral data collected at multiple sequential time points were analyzed using two-way repeated-measures ANOVA, followed by the Holm–Sidak post hoc multiple comparisons test. Statistical analysis was performed using GraphPad Prism, and results were considered statistically significant at *P* < 0.05.

## Results

### SARM1 expression increased in neurons of the peri-infarct cortex at an early stage after photothrombotic stroke

**Fig. 1:**
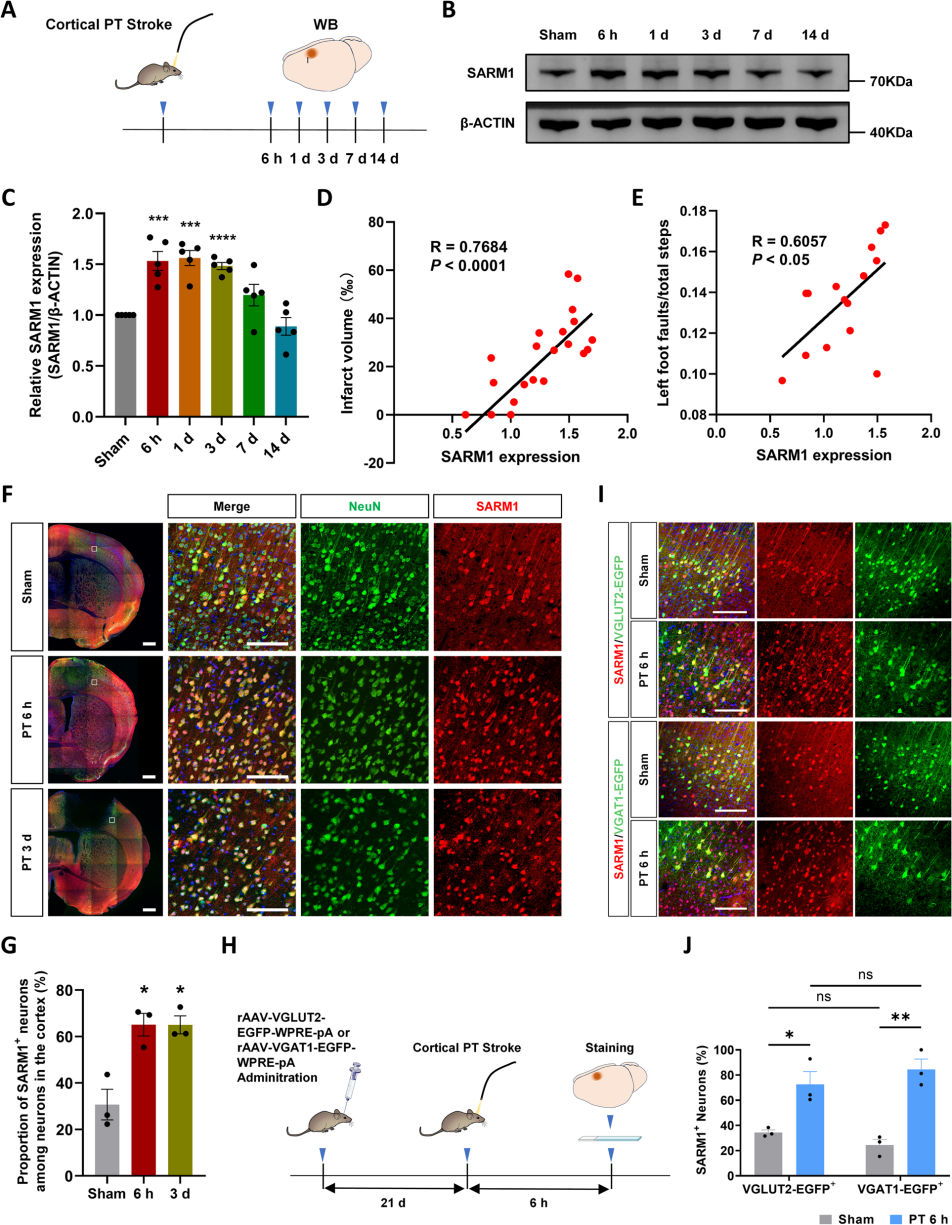
SARM1 was upregulated in neurons at an early stage after PTI**. A** Schematic of WB sampling. **B** WB analysis of SARM1 expression in the peri-infarct cortex at different stages after PTI. **C** Quantitative analysis of the relative SARM1 levels (normalized to sham group, n = 5 per group). **D**, **E** Correlation analysis of SARM1 expresion with the infarct volume and the behavioral outcome of PT stroke model. **F** Double immunofluorescent staining of SARM1 (green) and NeuN (red) in brain sections of sham-treated mice and mice at 6 h and 3 d after PTI. Nuclei were labeled with DAPI (blue). **G** Quantitative analysis of the proportion of SARM1 positive neurons of the cortex at different stages after PTI (n = 3). **H** Schematic of rAAV-VGLUT2-EGFP or rAAV-VGAT1-EGFP administration and PTI. **I** Immunofluorescence analysis showing SARM1^+^/EGFP^+^ cells in the cortex at 6 h after PTI. **J** Quantification of the proportion of SARM1^+^ neurons in all VGLUT2-EGFP^+^ and VGAT1-EGFP^+^ neurons in the cortex, respectively (n = 3). Scale bars, whole-mount images, 500 µm; higher-magnification images, 100 µm. Data were mean ± SEM. ^*^P < 0.05, ^**^P < 0.01, ^***^P < 0.001

To explore the function of SARM1 after stroke, we examined its expression pattern in the cortex at different time points after PTI. The level of SARM1 was significantly upregulated in the post-PTI group compared with that in the sham-treated group at 6 h post PTI, persisted until 3 d, and began to decline at 7 d (Fig. 1A-C). Moreover, the level of SARM1 positively correlated with the infarct volume and the neurological deficit (Fig. 1D and E). Subsequently, we investigated the cellular distribution of SARM1 using immunofluorescence staining after PTI. We observed clear colocalization of SARM1 and NeuN in both the sham-treated and stroke-injured groups (Fig. 1F), whereas no colocalization was detected between SARM1 and Iba1 or GFAP (Supplementary Fig. 2). Notably, not all neurons expressed SARM1. The percentage of SARM1-positive neurons increased at 6 h and 3 d post PTI (Fig. 1G).

To determine the specific neuronal subtypes expressing SARM1, we injected rAAV-VGLUT2-EGFP-WPRE-pA and rAAV-VGAT1-EGFP-WPRE-pA into the cortical region before PTI to label excitatory and inhibitory neurons, respectively (Fig. 1H). We found that 34.42% ± 2.02% of excitatory neurons and 24.36% ± 4.623% of inhibitory neurons expressed SARM1 (Fig. 1I and J), with no significant difference between the two groups. The proportion of both excitatory and inhibitory neurons expressing SARM1 increased at 6 h post PTI, with 72.56% ± 10.21% of excitatory neurons and 84.49% ± 8.18% of inhibitory neurons expressing SARM1 (Fig. 1I and J). No significant differences were observed between excitatory and inhibitory neurons. Collectively, these results indicate that SARM1 is expressed in neurons, upregulated at early stages after PTI, and evenly distributed between excitatory and inhibitory neurons under both physiological and stroke-induced pathological conditions.

### Deletion of SARM1 improved neurological function, reduced infarct volume and the inflammatory response

To address the role of SARM1 in cortical injury after PTI, *Sarm1*^−/−^ mice were obtained from Cyagen Biosciences Inc. The knockout efficiency of *Sarm1*^−/−^ mice was verified by qPCR and WB, and the results showed that the mRNA and protein levels of SARM1 were significantly suppressed (Supplementary Fig. 3A-C). We next examined whether SARM1 deletion affected growth and neurological function. As shown in Supplementary Fig. 3D, there was no significant difference in body weight between WT and *Sarm1*^−/−^ mice. Moreover, SARM1 deletion did not affect motor or sensor function, based on behavioral tests including the gridwalking test and the adhesive removal test (Supplementary Fig. 3E).

**Fig. 2:**
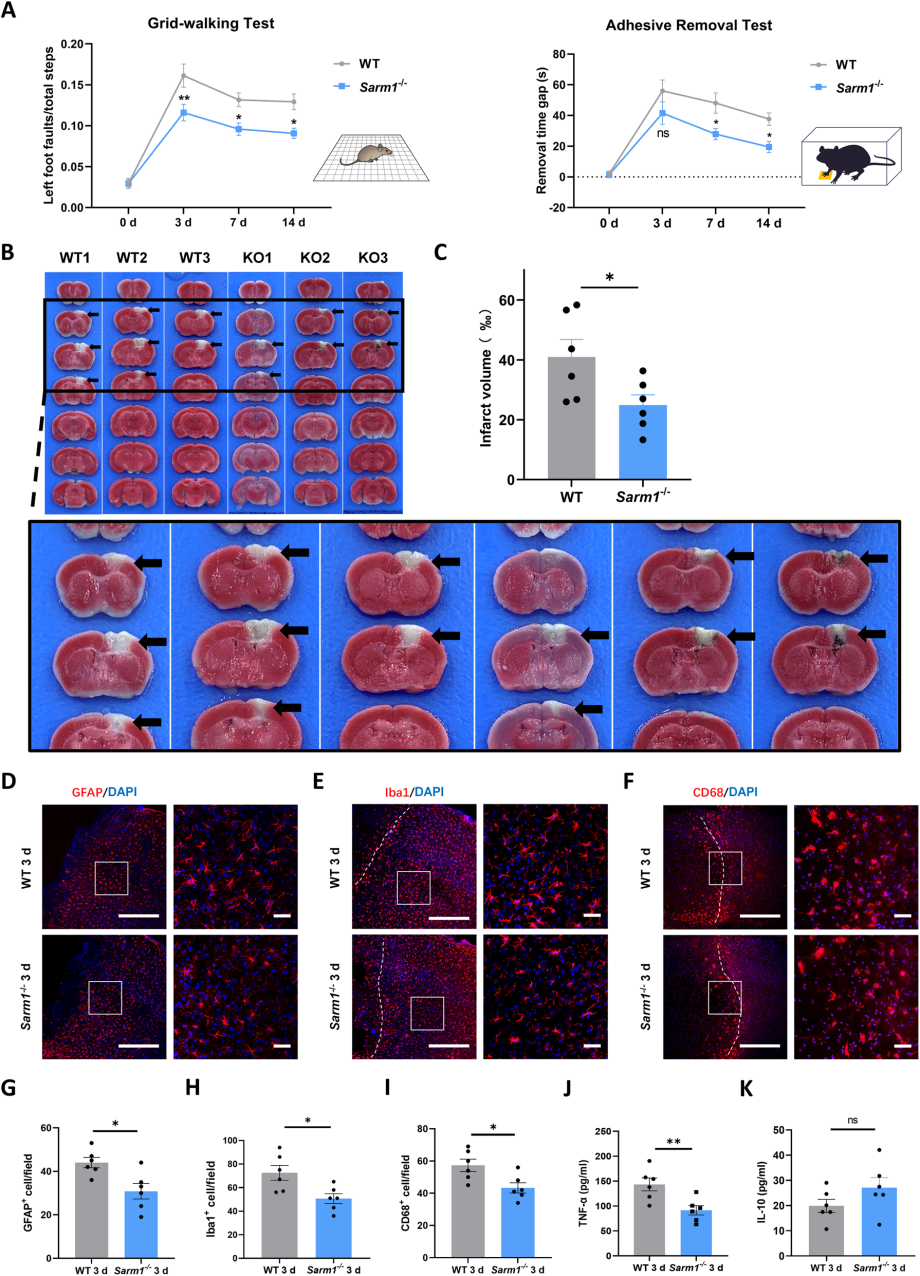
Deletion of Sarm1 improved neurological function, reduced infarct volume and the inflammatory response**. A** Neurological function of WT mice and *Sarm1*^−/−^ mice at baseline and 3d, 7d, 14 d were evaluated using the gridwalking test (n = 9 WT group, n = 10 *Sarm1*^−/−^ group) and the adhesive removal test (n = 10). Statistical analysis was performed by two-way repeated-measures ANOVA followed by Holm-Sidak post hoc multiple comparisons test. **B** Representative photographs of coronal brain sections of WT mice and *Sarm1*^−/−^ mice stained with TTC 3 d after PTI. **C** Quantitative analysis of infarct volume of WT mice and *Sarm1*^−/−^ mice (n = 6). **D**–**F** Immunostaining analysis of GFAP, Iba1, and CD68 in the peri-infarct cortex of WT and *Sarm1*^−/−^ mice at 3 d after PTI. **G**–**I** Quantitative analysis of the density of GFAP^+^ cells, Iba1^+^ cells and CD68^+^ cells (n = 6 per group). **J**, **K** ELISA analysis of TNF-α and IL-10 in brain tissue (n = 6). Dashed lines indicated the outline of the microglial accumulation band. Images of selected regions (rectangles) in (**D**), (**E**), and (**F**) were shown at higher magnification. Scale bars, whole-mount images, 500 µm; higher-magnification images, 100 µm. Data are presented as the mean ± SEM. ^*^P < 0.05, ^**^P < 0.01, ^***^P < 0.001

To determine the influence of SARM1 deletion on ischemic injury, *Sarm1*^⁻/⁻^ and WT mice were subjected to PTI. First, we tested whether SARM1 deletion affected behavioral outcomes after ischemic stroke. After PTI, mice showed substantial deficits in the grid-walking and adhesive removal tests, whereas SARM1 deletion improved performance at 3 d post ischemic stroke, and this advantage persisted for 14 d (Fig. 2A). The infarct volume peaked at 3 d (Supplementary Fig. 1B and C), consistent with a previous MRI report on a photothrombotic stroke model [2]. Subsequently, TTC staining was used to evaluate infarcted tissue volume after 3 d. The infarct volume in *Sarm1*^⁻/⁻^ mice was smaller than that in WT mice (*P* < 0.05, Fig. 2B and C). To further confirm the neuroprotective effect of SARM1 deletion in stroke-affected mice, WT and *Sarm1*^⁻/⁻^ mice were subjected to 60 min of ischemia followed by 24 h of reperfusion. TTC staining and Longa scores were used to evaluate infarct volume and neurological performance, respectively. The infarct volume in *Sarm1*^*⁻/⁻*^ mice was smaller than that in WT mice (*P* < 0.05, Supplementary Fig. 1D and E), and the neurological function score was lower in *Sarm1*^⁻/⁻^ mice (Supplementary Fig. 1F).

Following TTC staining demonstrating reduced brain injury at 3 days post stroke, we further explored the effect of SARM1 deletion on the local tissue inflammatory response. We found that SARM1 deletion reduced the accumulation of astrocytes, the accumulation as well as activation of microglia in the peri-infarct cortex (Fig. 2D-I). Meanwhile, inflammation-related cytokine levels were detected by ELISA and a significant decrease in TNF-α was noticed (Fig. 2J and K). 

Collectively, these results suggest that SARM1 deletion improves neurological outcomes, reduces infarct volume and the inflammatory response around the infarction.

### Peri-infarct neurons and NAD⁺ were preserved and SIRT1 was elevated in Sarm1^⁻/⁻^ mice

**Fig. 3:**
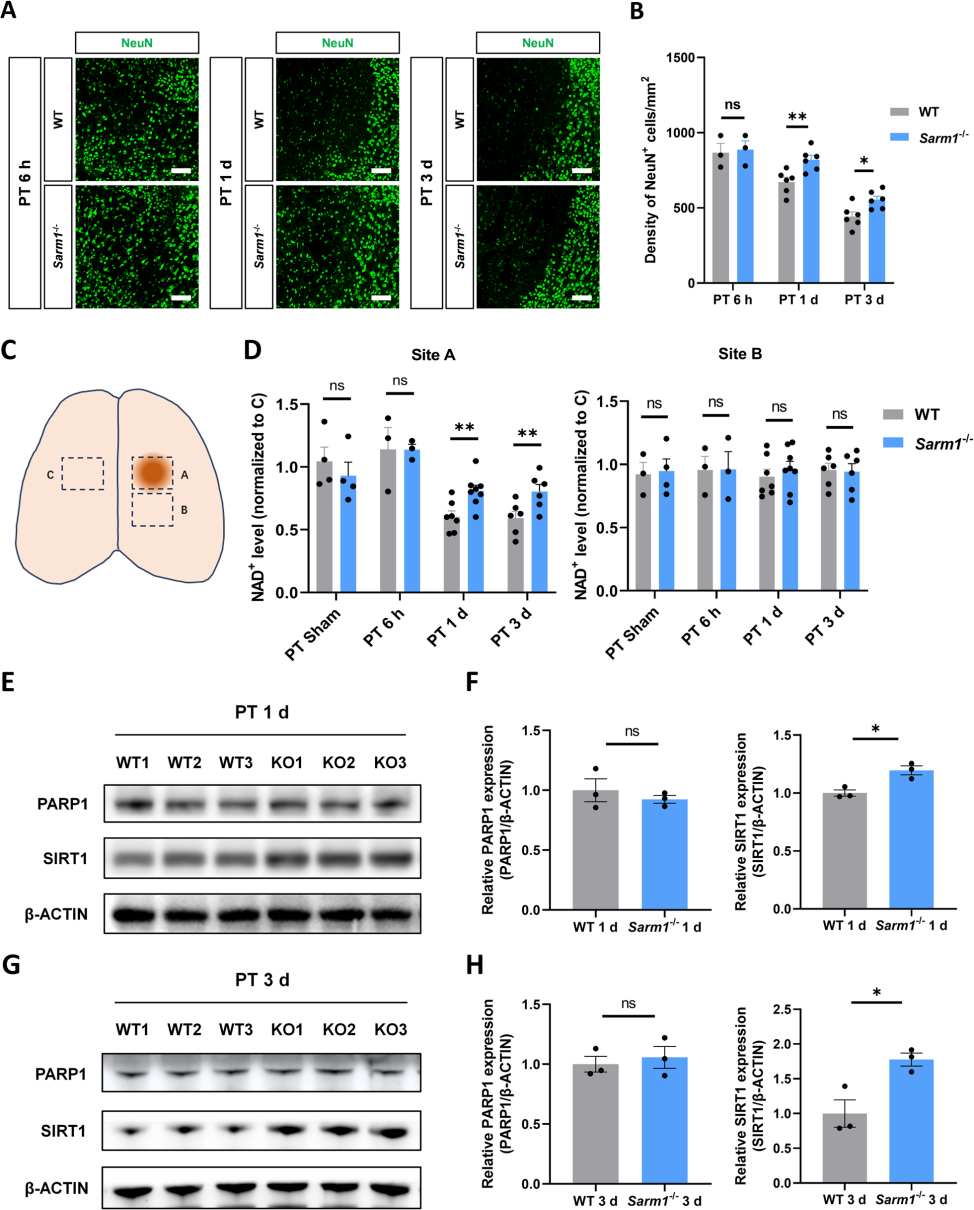
Peri-infarct neurons and NAD⁺ were preserved and SIRT1 was elevated in *Sarm1*^⁻/⁻^ mice. **A** Immunofluorescence staining of NeuN (green) in WT mice and *Sarm1*^−/−^ mice and **B** quantitative analysis of neuron counts. **C** Schematic diagram of tissue collection for NAD^+^ detection. **D** The NAD^+^ levels at site A and B of WT and *Sarm1*^−/−^ mice peri-infarct cortex at 6 h, 1 d and 3 d after PTI, normalized to the NAD^+^ level at site C. WB analysis of the expression of PARP1 and SIRT1 in the peri-infarct cortex of WT and *Sarm1*^−/−^ mice at 1 d **E** and 3 d **G** after PTI. Quantitative analysis of the relative PARP1 and SIRT1 levels at 1 d **F** and 3 d **H** after PTI (n = 3 per group). Data are presented as the mean ± SEM. Scale bars, 100 µm. ^*^P < 0.05, ^**^P < 0.01, ^***^P < 0.001

To investigate how SARM1 deletion influenced focal infarction in the cortex, we quantified the number of neurons in the peri-infarct area using immunofluorescence staining with NeuN. Microglial cells were simultaneously stained with Iba1 to identify the peri-infarct area. The number of surviving neurons in *Sarm1*^⁻/⁻^ mice did not differ significantly from that in WT mice at 6 h post PTI but was significantly increased at 1 d and 3 d post PTI (Fig. 3A and B).

To explore the mechanism underlying neuronal preservation in *Sarm1*^⁻/⁻^ mice, NAD⁺ levels in the cortical tissue post PTI were measured. The infarcted cortex was collected as shown in the schematic (Fig. 3C). The NAD⁺ level at site C was used as the control for each mouse. NAD⁺ levels at the three sites were measured at 6 h, 1 d and 3 d post PTI. There was no significant difference in NAD⁺ levels between stroke and sham-treated mice at site B (Fig. 3D). The NAD⁺ level at site A did not vary at 6 h post PTI but was significantly decreased at 1 d. Notably, *Sarm1*^⁻/⁻^ mice exhibited better NAD⁺ preservation than WT mice at 1 d and 3 d post PTI (Fig. 3D).

Further exploration of NAD^+^ metabolism pathways was performed. The time points when NAD^+^ concentration differed significantly post PTI were chosen to detect NAD^+^-related molecules. We found that there was a difference in the level of sirtuin 1 (SIRT1) in the peri-infarct tissue between WT and *Sarm1*^⁻/⁻^ mice. The SIRT1 level was significantly increased in *Sarm1*^⁻/⁻^mice 1 d after PTI (Fig. 3E and F). The SIRT1 level remained elevated in *Sarm1*^⁻/⁻^ mice 3 d after PTI, which showed a more pronounced trend (Fig. 3G and H). Furthermore, there was no significant difference in the level of poly(ADP-ribose) polymerase 1 (PARP1) between the two groups (Fig. 3E–H).

Collectively, these results suggest that neurons lacking SARM1 are better preserved after ischemic injury, likely because of reduced NAD⁺ consumption. SARM1 deletion also increases the expression of SIRT1 after PTI, which could be associated with NAD⁺ preservation and the inhibition of inflammatory response.

### Protective effect of SARM1 deletion on axonal degeneration in the internal capsule (IC) and cerebral peduncle (CP) at 3 d post PTI

**Fig. 4:**
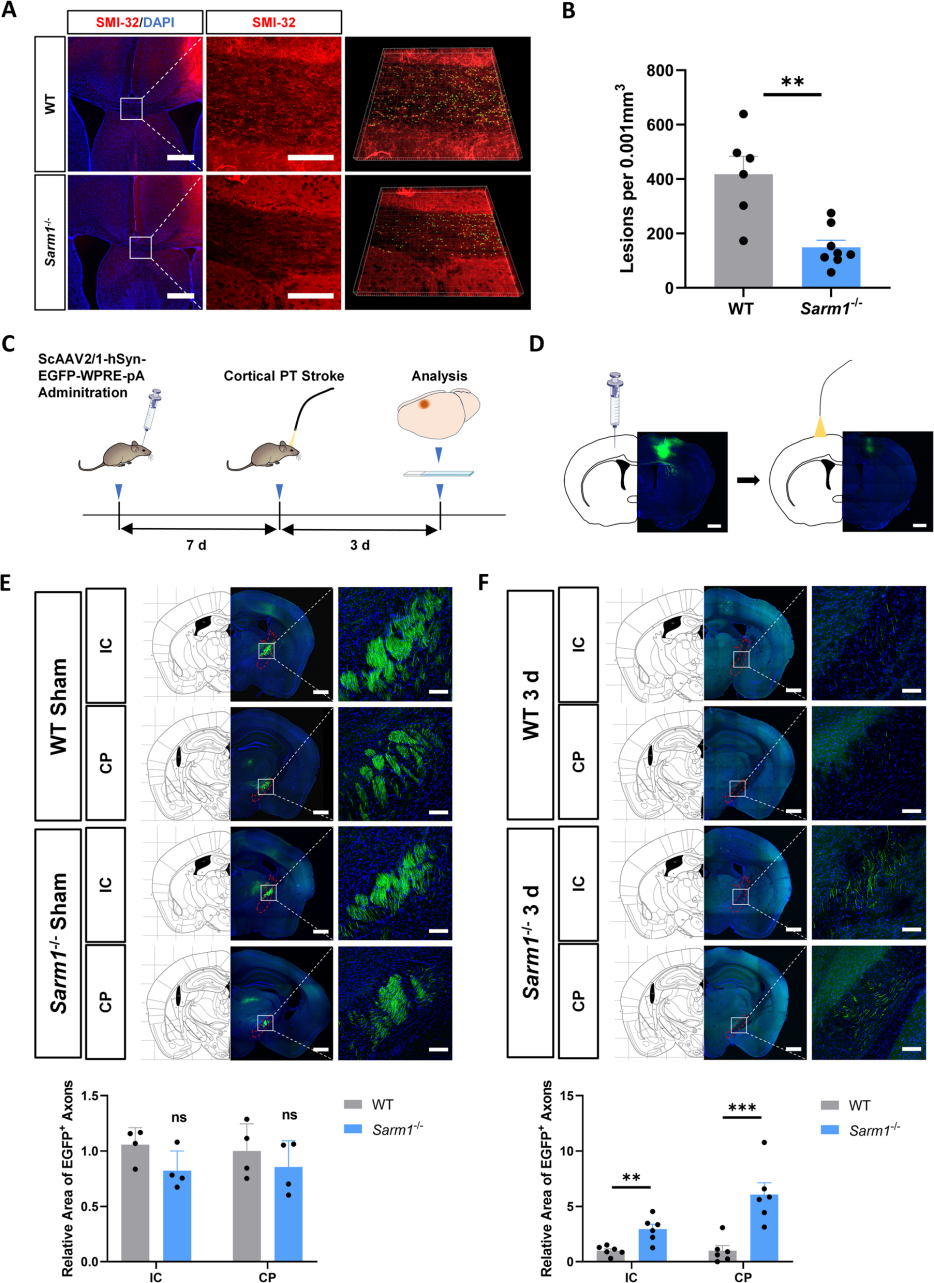
Protective effect of SARM1 deletion on axonal degeneration in the IC and CP at 3 d post PTI. **A** Immunofluorescence analysis of SMI-32 (red) in the CC at 3 d after PTI. Nuclei were labeled with DAPI (blue). **B** Quantitative analysis of SMI-32 labeled axons and spheroids (n = 6 WT mice, n = 8 *Sarm1*^−/−^ mice). **C** Schematic of ScAAV-hSyn-EGFP administration and PTI. **D** Representative whole-mount photograph of brain slices after ScAAV-hSyn-EGFP administration and PTI. **E** Labeled axons in IC and CP after ScAAV-hSyn-EGFP administration and sham operation (n = 4). **F** Labeled axons in IC and CP after ScAAV-hSyn-EGFP administration and PTI (n = 6). Relevant brain regions are highlighted. Scale bars, whole-mount images, 500 µm; higher-magnification images, 100 µm. Data are presented as the mean ± SEM. ^*^P < 0.05, ^**^P < 0.01, ^***^P < 0.001

To examine the influence of SARM1 deletion on axonal injury, brain slices from mice post PTI were stained with SMI-32 to identify non-phosphorylated neurofilaments. The accumulation of SMI-32 has been used as a marker of axonal injury [30]. Axonal damage and spheroid formation in the corpus callosum (CC) were alleviated in *Sarm1*^⁻/⁻^ mice (*P* < 0.01, Fig. 4A and B).

To investigate how SARM1 deletion affects behavioral function post PTI, we designed the following experiment (Fig. 4C). Cortical neurons were labeled with ScAAV-hSyn-EGFP-WPRE-pA (Fig. 4D). Seven days after AAV administration, enhanced green flourescent protein (EGFP) synthesized in cortical motor neurons was transported to the brainstem and spinal cord via axons, labeling part of the corticospinal tract. EGFP⁺ axons projecting to the IC and CP were observed in coronal brain slices, and EGFP⁺ axon areas were quantified. No difference was observed between sham-treated WT and *Sarm1*^⁻/⁻^ mice in EGFP⁺ axon areas in either the IC or CP (Fig. 4E). Three days later, infected neurons were damaged by PTI (Fig. 4D). Most EGFP⁺ axons degenerated, but the remaining fragmented axonal areas in the IC (*P* < 0.01) and CP (*P* < 0.001) were significantly larger in *Sarm1*^⁻/⁻^ mice than in WT mice (Fig. 4F). Collectively, these results suggest that SARM1 deletion exerts a protective effect against axonal degeneration at 3 d post PTI.

### SARM1 deficiency inhibited glial scar formation and decreased activated microglia at 2 weeks after PTI

**Fig. 5:**
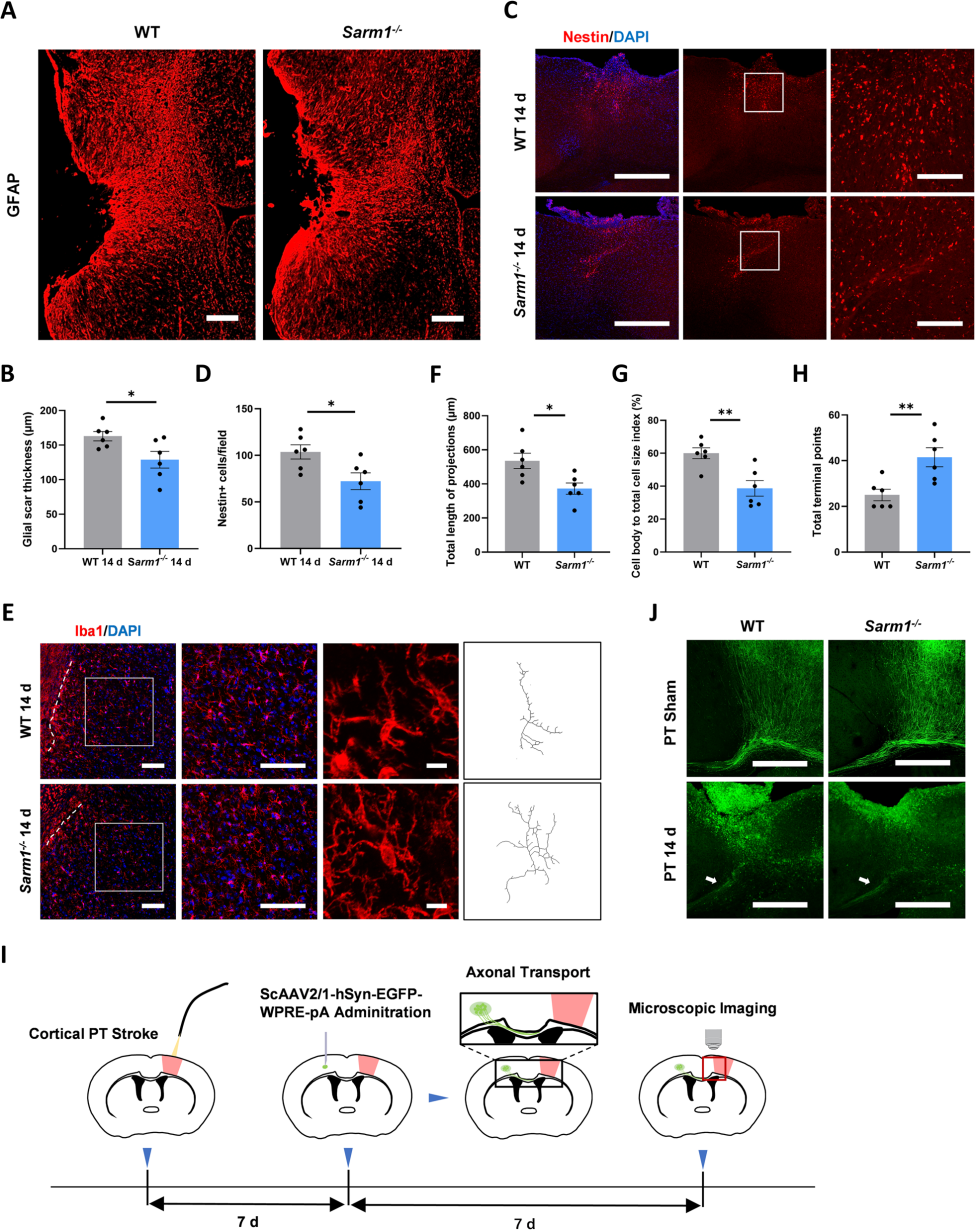
SARM1 deficiency inhibited glial scar formation and decreased activated microglia at 2 weeks after PTI. **A** The representative glial scar images of WT mice and *Sarm1*^−/−^ mice with GFAP immunofluorescent staining. Scale bars, 100 µm. **B** The quantitation of glial scar thickness. n = 6. **C** Representative images of cells stained with Nestin within the glial scar. Scale bars, whole-mount images, 500 µm; higher-magnification images, 100 µm. **D** The quantitation of the Nestin^+^ cell density. n = 6. **E** Immunofluorescence staining of Iba1. Dashed lines indicated the outline of the microglial accumulation band. Black lines showed the skeleton of microglia. Scale bars, whole-mount images and higher-magnification images, 100 µm; sholl-analysis images, 5 µm. **F** Quantitative analysis of the total length of projections. n = 6. **G** Quantitative analysis of the cell body to total cell size index. n = 6. **H** Quantitative analysis of the total terminal points. n = 6. **I** Schematic diagram of experimental design. **J** Representative images of GFP-labeled axons (green fibers). The white arrows point to the damaged axonal terminals. Scale bar, 500 µm. Data are presented as the mean ± SEM. ^*^P < 0.05, ^**^P < 0.01, ^***^P < 0.001

Given the sustained behavioral improvement in *Sarm1*^⁻/⁻^ mice at 14 days, histological analysis of the peri-infarct zone was performed. First, we found that SARM1 deletion markedly reduced the thickness of GFAP-labeled glial scars (Fig. 5A and B). Nestin is a marker of neural precursor cells. Reactive astrocytes re-express Nestin and participate in the initial formation of glial scars. In our experiment, we found a decrease in Nestin-positive cells at the glial scar site in *Sarm1*^⁻/⁻^ mice (Fig. 5C and D).

Alterations in microglial morphology suggest changes in microglial function [20]. We stained microglia cells with Iba1, and evaluated the effects of SARM1 deletion on microglial morphology in the peri-infarct cortex using Image J. Stroke injury caused deramification of microglia, which was defined as shortened projections and enlarged cell bodies. We found that SARM1 deletion increased the total length of these projections, the total terminal points, and decreased the cell body-to-total cell size index (Fig. 5E-H).

The neurofunctional advantage of *Sarm1*^⁻/⁻^ mice persisted till 2 weeks, prompting us to speculate whether SARM1 is involved in the process of axonal regeneration, which has not been mentioned in previous studies. We therefore designed the following experiment, details of which are shown in the schematic diagram (Fig. 5I). According to the experimental design, neurons infected with ScAAV express EGFP, which is gradually transported along axons. In the sham group, we observed that EGFP expressed by infected contralateral neurons in both groups of mice was transported along axons to the ipsilateral cortex. If the axons regenerated, the newly formed axons could be visualized by EGFP labeling in the infarcted cortex. However, as shown in Fig. 5J, the labeled axons after PTI in both groups only extended to the CC around the infarction, suggesting no sign of axon regeneration.

In summary, we found that SARM1 deletion inhibits the formation of glial scar, and simultaneously alleviates the activation of microglia in the peri-infarct cortex at 2 weeks after PTI.

### FK866 and DSRM-3716 failed to alleviate cortical injury in mice

**Fig. 6:**
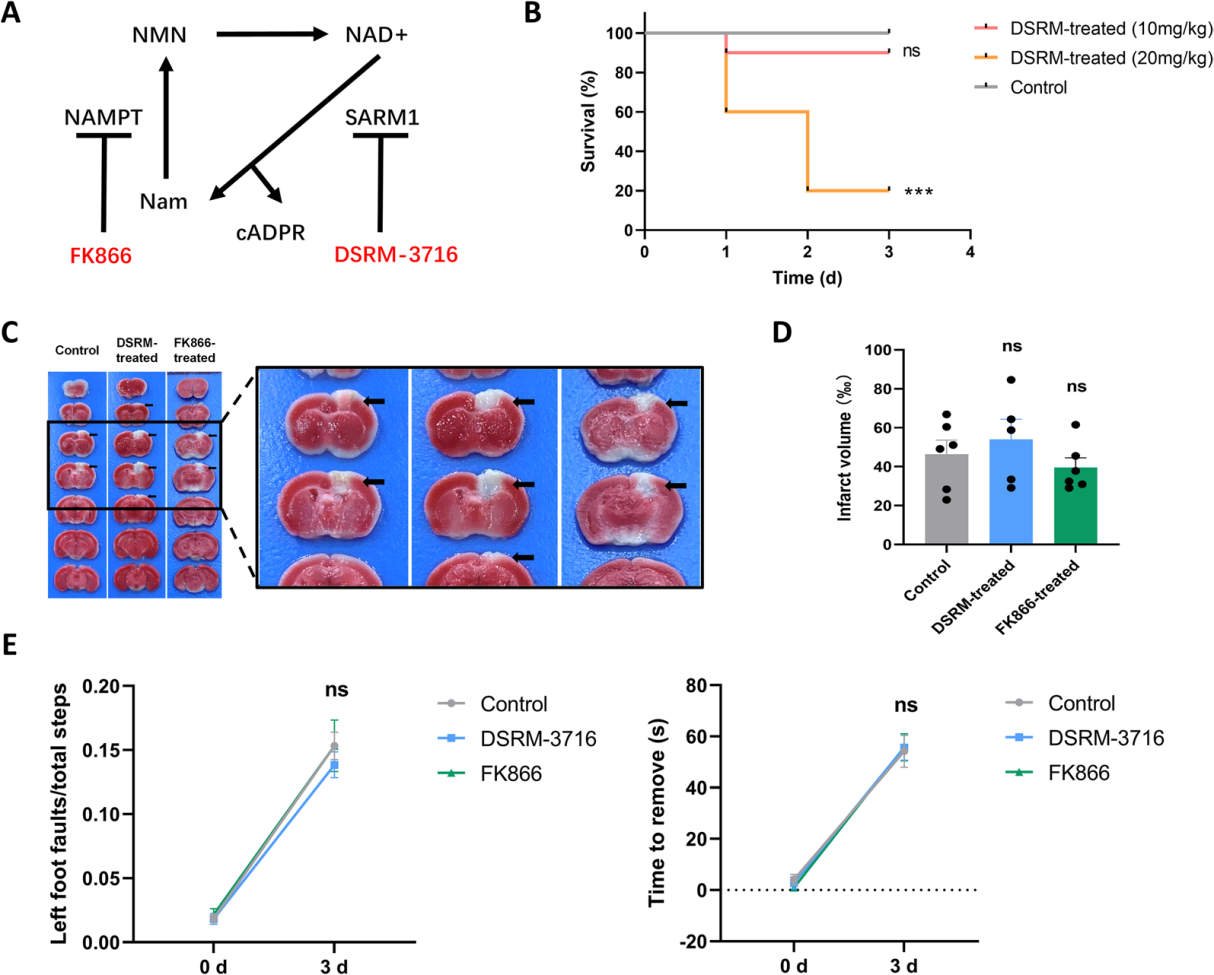
FK866 and DSRM-3716 failed to alleviate cortex injury in mice. **A** Schematic diagram of NAD^+^ pathway and pharmacological inhibitors used. **B** Survival statistics of the mice post PTI in control group, DSRM-treated (10mg/kg) group and DSRM-treated (20mg/kg) group (n = 10). **C** Representative photographs of coronal brain sections of mice stained with TTC 3 d after PTI treated with solvent, DSRM-3716 and FK866. **D** Quantitative analysis of infarct volume as shown in **C** (n = 6 control group, n = 5 DSRM-treated group, n = 6 FK866-treated group). **E** Neurological function of solvent-treated, DSRM-treated and FK866-treated mice at baseline and 3 d evaluated using the grid walking test (n = 14 control group, n = 13 DSRM-treated group, n = 12 FK866-treated group) and the adhtesive removal test (n = 10 control group, n = 10 DSRM-treated group, n = 12 FK866-treated group). Data are presented as the mean ± SEM. ^*^P < 0.05, ^**^P < 0.01, ^***^P < 0.001

To explore the translational value of this study for patients with stroke, we examined whether inhibition of the SARM1 pathway alleviated cortical injury and improved neurological performance after PTI (Fig. 6A). FK866, a feedback inhibitor of SARM1 that increases nicotinamide levels in neurons [7], was administered intraperitoneally to mice after PTI. As shown in Fig. 6C–E, there was no significant difference in infarct volume or behavioral performance between FK866-treated and control mice. A potent and selective small-molecule isoquinoline inhibitor of SARM1, DSRM-3716, has been reported to protect axons from degeneration in vitro [15]. The DSRM-treated (10 mg/kg) and control groups were subjected to PTI. The SARM1 inhibitor did not significantly reduce infarct volume or improve neurological outcomes (Fig. 6C–E). To further assess its effects, DSRM-3716 was administered at a dose of 20 mg/kg daily for 3 d post PTI. The survival rate of stroke-affected mice decreased sharply after 3 d of administration (Fig. 6B). The results suggest that these two recently reported pharmacological inhibitors of SARM1 failed to mitigate brain injury in mice with photothrombotic stroke.

## Discussion

Our results show that the loss of SARM1, which exerts a pro-neurodegenerative function through NADase activity, substantially attenuates neuronal injury and axonal degeneration after acute ischemic stroke. At 1 d and 3 d, the number of NeuN⁺ cells in the peri-infarct area was increased, and NAD⁺ concentration was better maintained in *Sarm1*^⁻/⁻^ mice. Importantly, *Sarm1*^⁻/⁻^ mice exhibited substantial preservation of neurological function for up to 14 d post stroke, indicating that SARM1 loss is neuroprotective rather than merely delaying neuronal and axonal death. These findings provide a rationale for developing anti-SARM1 therapies to protect against brain injury in patients with stroke. However, FK866 and DSRM-3716, recently reported pharmacological inhibitors of SARM1, failed to replicate the *Sarm1*^⁻/⁻^ phenotype in a photothrombotic stroke model.

The role of SARM1 in central nervous system diseases, particularly acute injury, has been increasingly recognized. In a traumatic brain injury model, SARM1 knockout reduced the accumulation of β-amyloid precursor protein in the CC and phosphorylated NFH in blood, with neurological function scores better preserved in knockout mice than in WT mice [11]. In a mouse model of traumatic axonal injury, SARM1 deletion significantly reduced axonal lesions 24 h post injury [38]. Huitao Liu et al. found that SARM1 was upregulated in neurons and astrocytes early after spinal cord injury, and both SARM1^Nestin^-CKO and SARM1^GFAP^-CKO mice exhibited reduced neuroinflammation via downregulation of NF-κB signaling and enhanced neuronal regeneration [23]. Wallerian degeneration involves a self-destructive process that results in rapid NAD⁺ depletion and fragmentation of the distal portion of a severed axon [17]. Critically, complete axonal transection is irreversible, leading to degeneration of the distal axon. However, axonal damage in neurological diseases does not occur immediately, suggesting that NAD⁺ preservation may protect axons from degeneration. Henninger et al. observed neurological improvement in *Sarm1*^⁻/⁻^ mice within 24 h of TBI induction, in which damaged axons underwent secondary injury over hours to days after the initial trauma [11]. This has important implications for stroke treatment, as ischemia initiates a cascade of pathological events leading to neuronal injury at different time points during the acute phase.

A study by Xue et al. found that a SARM1 serine-548-alanine mutation in an MCAO/R model reduced infarct volume, decreased neuronal death, and improved behavioral performance [35]. Consistently, our results showed that *Sarm1*^⁻/⁻^ mice had smaller infarct volumes, better preserved neurons in the peri-infarct area, and superior neurological function in stroke models. Furthermore, we found that *Sarm1*^⁻/⁻^ mice exhibited NAD⁺ preservation and upregulated SIRT1 level in the peri-infarct cortex, suggesting a potential mechanism for neuronal protection. Pyramidal neurons were labeled using ScAAV, demonstrating a correlation between the neuroprotective effect of SARM1 deletion and improved fine motor function. We also found that SARM1 deletion inhibited neuroinflammation at 3 days, inhibited the formation of glial scar and simultaneously alleviated the activation of microglia at 14 days. Finally, we tested the effects of SARM1 inhibitors in stroke-affected mice to assess their translational value.

During the acute phase of stroke, the behavioral improvements observed in *Sarm1*^⁻/⁻^ mice post PTI may be attributed to the neuroprotective effects of SARM1 deletion. Our results demonstrated that SARM1 expression increased in neurons of the peri-infarct cortex at 6 h post PTI. As the infarct progressed, peri-infarct neuronal survival was significantly higher in *Sarm1*^⁻/⁻^ mice at 1d and 3d. NAD⁺ is a key coenzyme in mitochondrial oxidative phosphorylation and glycolysis and plays a critical role in ATP production. ATP depletion impairs neuronal ion homeostasis, leading to water influx from the extracellular space and neuronal swelling [6]. NAD⁺ depletion, independent of PARP1 activation, results in glycolysis inhibition, mitochondrial depolarization, apoptosis-inducing factor translocation, and neuronal death [1]. Our results align well with this finding and extend it in a way. *Sarm1*^⁻/⁻^ mice exhibited reduced neuronal death and preserved NAD^+^ in peri-infarct brain tissue, in which the PARP1 level remained unchanged. This suggests SARM1 mediates post-stroke neuronal death primarily through regulating NAD^+^ metabolism rather than PARP1 activation, providing new insights into targeting SARM1 to protect neurons. Zhao et al. found that NAD⁺ improved cognitive function and reduced neuroinflammation in chronic cerebral hypoperfusion models by suppressing reactive oxygen species production from damaged microglial mitochondria in the cortex and hippocampus via the Sirt1/PGC-1α pathway [37]. Our findings show that SARM1 deletion reduced neuronal death, preserved NAD^+^, and significantly elevated SIRT1 level in peri-infarct brain tissue, which suggests SARM1 deletion activates the SIRT1 pathway to reduce neuroinflammation and to protect neurons, highlighting a novel endogenous mechanism for neuroprotection. These results sufficiently indicate that the advantage of increased neurons in *Sarm1*^⁻/⁻^ mice post PTI is related to NAD⁺ preservation in neurons. Chiao-Po Hsum et al. found that cellular NAD⁺ was significantly depleted in cardiac myocytes as a result of myocardial ischemia in vivo [12]. None of the previous studies have reported NAD⁺ detection in stroke models. Our results first revealed that NAD⁺ concentration decreased in the peri-infarct cortex, and *Sarm1*^⁻/⁻^ mice exhibited pronounced NAD⁺ preservation.

The axons of EGFP⁺ neurons in the motor cortex are incorporated into the corticospinal tract, which projects to the contralateral side of the brainstem and spinal cord, thereby controlling contralateral forelimb movement [10]. We found that *Sarm1*^⁻/⁻^ mice had more labeled axons in the corticospinal tract 3 d after PTI than those of WT mice, as observed in both the IC and CP on coronal brain sections. This finding explains how the protective effect of SARM1 deletion on neurons contributes to improved contralateral limb motor function in mice. The infarct area in the widely used MCAO/R mouse model includes the cortex, striatum, midbrain, and other regions supplied by the middle cerebral artery, making it difficult to establish a direct relation between neuronal injury and behavioral outcomes. By contrast, the photothrombotic stroke model produces infarction confined to the cortex. Thus, the observed improvements in fine sensory and motor function of a single upper limb were most likely due to reduced injury in the cortical neurons responsible for controlling that limb. Furthermore, by labeling the axons projecting from the contralateral cortical neurons to the injured cortex, we confirmed that there was no axon regeneration at 2 weeks after model establishment, which is also attributed to the advantage of the photothrombotic model in studying neuronal projections.

FK866 is a feedback inhibitor of SARM1 that has been widely used to limit nicotinamide consumption by inhibiting NAMPT in mouse models of central nervous system injury. Nikolaos K. Ziogas et al. reported a significant reduction in the number of axonal lesions 1 d after traumatic axonal injury when SARM1 was inhibited by FK866 [38]. Similarly, Huitao Liu et al. found that FK866 reduced neuroinflammation and facilitated neuronal regeneration following spinal cord injury [23]. However, in our study, FK866 failed to exert a protective effect in the photothrombotic stroke model at the administered dose. One of the putative reasons may be related to model-specific pathological features. Previous models (e.g., spinal cord injury) often involve more extensive and thorough neural damage, whereas the photothrombotic model primarily induces focal cortical ischemia with limited damage scope. The brain, particularly the cerebral cortex, features a complex microenvironment, diverse cellular composition, and extensive network connectivity—traits that distinguish it from other neural tissues. This could also explain the ineffectiveness of FK866. DSRM-3716 is a potent and selective small-molecule isoquinoline inhibitor of SARM1 NADase that protects axons from degeneration and rescues injured axons that have entered the metastable state because of rotenone exposure [15]. However, its application in central nervous system diseases in vivo has not been previously reported. We began administering DSRM-3716 to mice at a dose of 10 mg/kg once daily for three consecutive days after PTI. No protective effects were observed on infarct volume or behavioral performance. When the dose was doubled in an attempt to counteract stroke-induced deficits, the survival rate of the mice declined significantly compared with that of the control group. No additional experiments were conducted with alternative dosing regimens. Possible reason for ineffectiveness is that its in vitro axon-protective activity may not be applicable to the intricate pathological pathway in vivo. The high-dose mortality further suggests unrecognized toxicity in vivo, e.g., off-target effects or metabolic toxicity. It was reported that isoquinolines exhibited hepatotoxicity in a previous study [24]. The adverse reaction may negate efficacy to some extent. The pharmacokinetics or dosage window for mice should be optimized before applied to stroke models. Another possible reason for the poor efficacy of these two pharmacological inhibitors of SARM1 is the difficulty in achieving sufficient drug delivery across the blood–brain barrier. The blood–brain barrier prevents the inhibitors from reaching ischemic neurons, which are largely excluded from the brain following intraperitoneal administration [29]. Further research is required to explore the neuroprotective potential of other SARM1 inhibitors.

This study has some limitations. First, SARM1 cannot be effectively stained with primary antibodies in the infarct core, making it challenging to analyze its expression pattern in core-zone cells and its association with neuronal loss. Second, there are pathophysiological differences between rodent models and humans, and current models may not fully replicate the complexity of human stroke. Additionally, although SARM1 deletion significantly reduced infarct volume and inflammatory response, the specific mechanisms underlying the associated inflammatory changes remain unclear, limiting the ability to fully recapitulate the *Sarm1*^⁻/⁻^ phenotype in a photothrombotic stroke model.

In summary, our study identified the role of SARM1 in stroke pathophysiology. SARM1 expression increases in neurons of the peri-infarct cortex after PTI and genetic deletion of SARM1 protects neurons and inhibits axonal degeneration. Most importantly, SARM1 deficiency improves the mice’s neurological performance following PTI. Therefore, the SARM1 signaling pathway may serve as a promising therapeutic target for stroke treatment.

## Supplementary Information

Below is the link to the electronic supplementary material.

Supplementary Material 1

Supplementary Material 2

Supplementary Material 3

Supplementary Material 4

## Data Availability

All methods and materials used are described in the manuscript and data and analysis can be obtained from the corresponding or first author upon reasonable request.
