# Supplementary material for: Deficiency of SARM1 attenuates neuronal injury and improves neurological performance in a photothrombotic stroke model
Source: Mol Brain. 2025 Nov 21;18:87. doi: 10.1186/s13041-025-01251-5 (PMC12639794; doi:10.1186/s13041-025-01251-5)
Supplement: Supplementary file 1 — Supplementary Material 1 [file 13041_2025_1251_MOESM1_ESM.docx]

**Supplemntary figure 1.** (A) The schematic illustration of photothrombotic stroke model. The white dashed circle indicates the thinned area of skull. (B) Representative photographs of coronal brain sections from WT mice stained with TTC at 6 h, 1 d, 3 d, 7 d, and 14d after PTI and (C) quantitative analysis of infarct volume. (D) Representative photographs of coronal brain sections from *Sarm1*^-/-^ and WT mice stained with TTC at 24 h after MCAO/R and (E) the relative infarct volume (n = 6). (F) Neurological scores of *Sarm1*^-/-^ and WT mice at 24 h after MCAO/R. Data are presented as the mean ± SEM. ^*^*P* < 0.05, ^**^*P* < 0.01, ^***^*P* < 0.001.

**Supplemntary figure 2.** Double immunofluorescent staining of SARM1 (green) and Iba1/GFAP (red) in brain sections from sham-treated mice and mice at 6 h after PTI. Nuclei were labeled with DAPI (blue). Scale bars, whole-mount images, 500 µm; higher-magnification images, 100 µm.

**Supplemntary figure 3.** (A) The mRNA abundance of *Sarm1* in the cortex of *Sarm1^-/-^* mice normalized to that of WT mice (n=3). (B) WB analysis of SARM1 expression in the cerebral cortex and spinal cord of WT mice and *Sarm1^-/-^* mice. (C) Quantitative analysis of the relative SARM1 levels of *Sarm1^-/-^* mice normalized to those of WT mice (n = 3). (D) Quantitative analysis of the body weight of adult WT mice and *Sarm1^-/-^* mice (n = 10). (E) Neurological function of WT mice and *Sarm1^-/-^* mice were evaluated using the grid-walking test and the adhesive removal test (n = 10). Data are presented as the mean ± SEM. ^*^*P* < 0.05, ^**^*P* < 0.01, ^***^*P* < 0.001.
