# Supplementary figures and images for: Deficiency of SARM1 attenuates neuronal injury and improves neurological performance in a photothrombotic stroke model
Source: Mol Brain. 2025 Nov 21;18:87. doi: 10.1186/s13041-025-01251-5 (PMC12639794; doi:10.1186/s13041-025-01251-5)

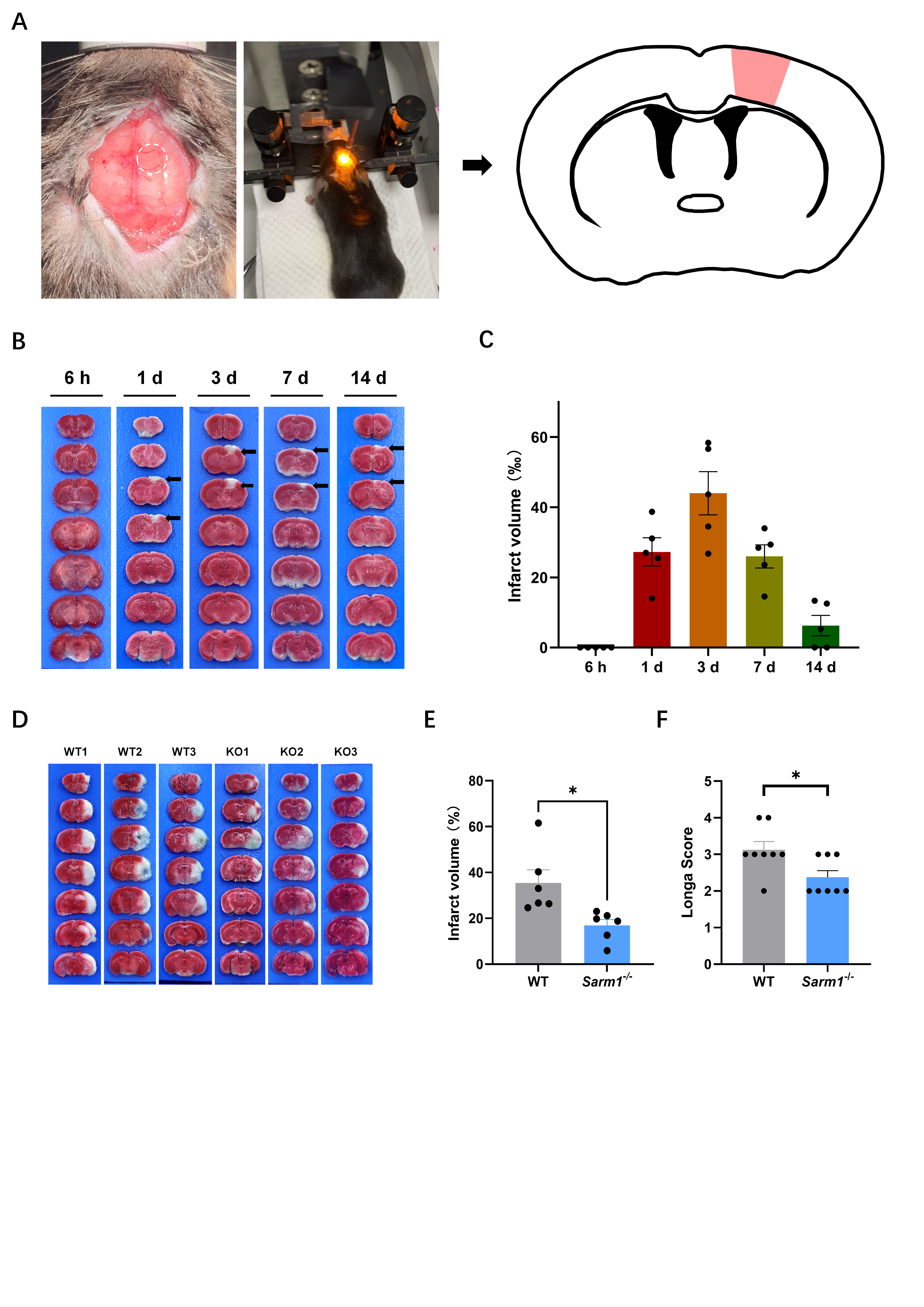

Supplement: Supplementary file 2 — Supplementary Material 2 [file 13041_2025_1251_MOESM2_ESM.tif]

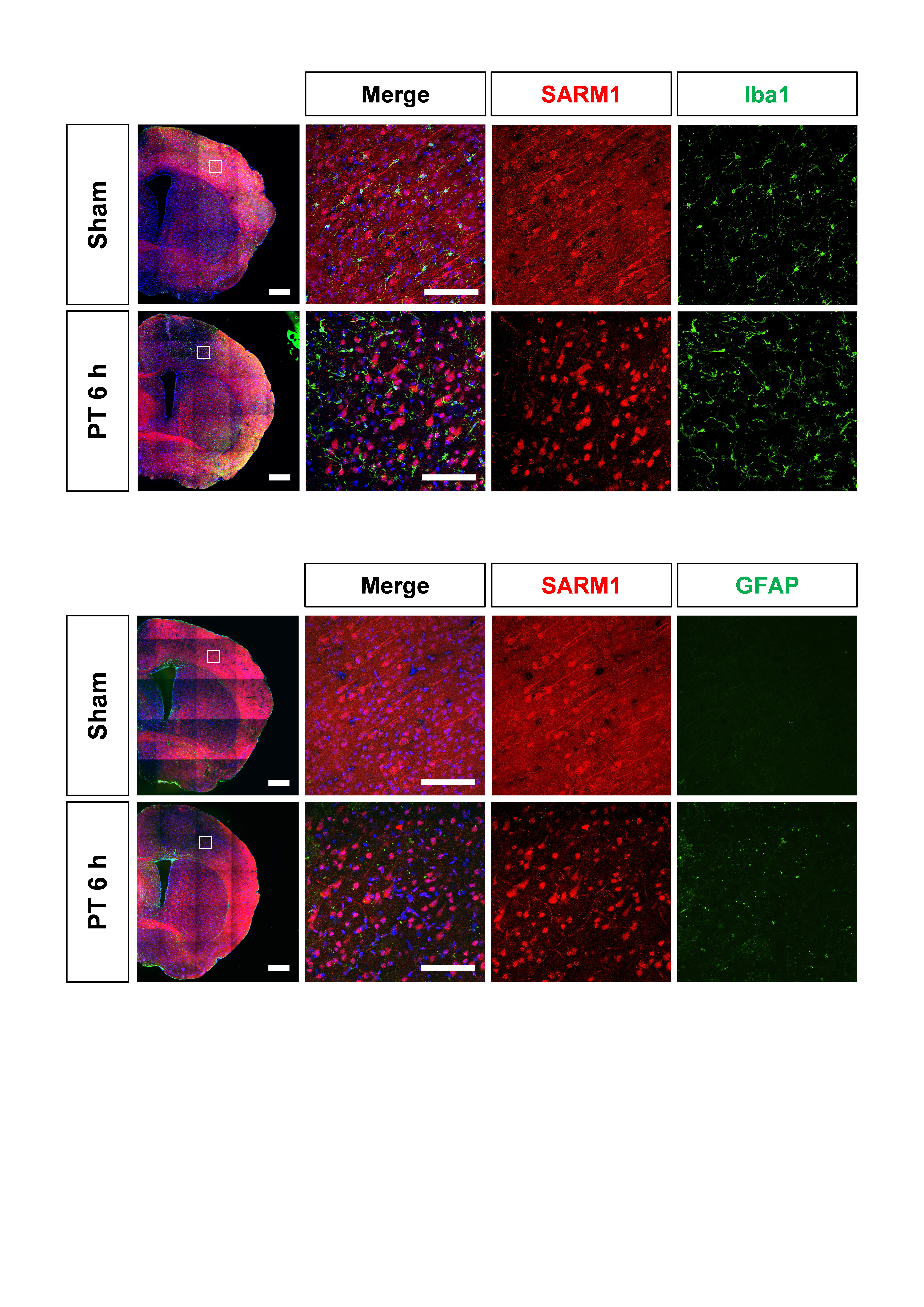

Supplement: Supplementary file 3 — Supplementary Material 3 [file 13041_2025_1251_MOESM3_ESM.tif]

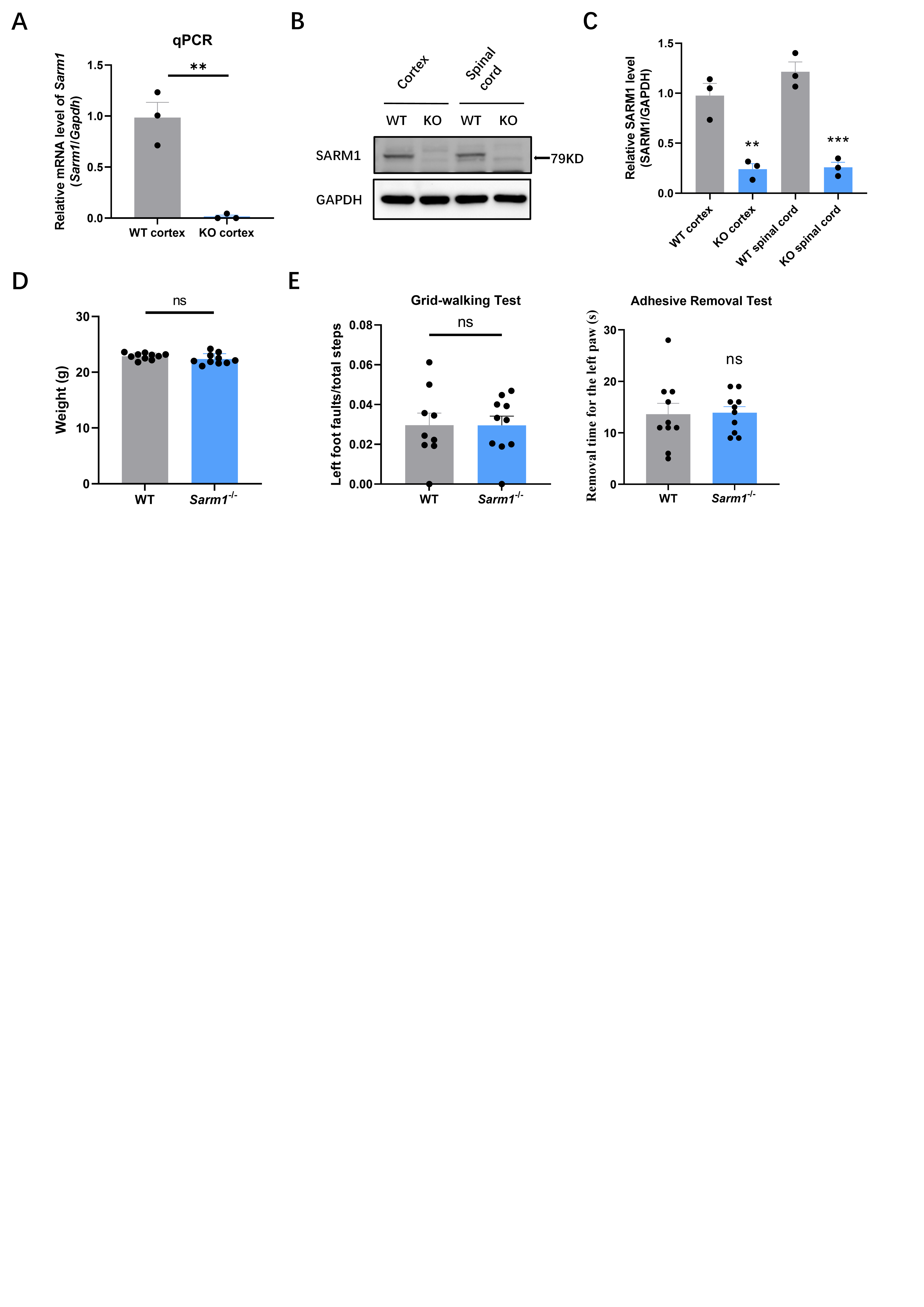

Supplement: Supplementary file 4 — Supplementary Material 4 [file 13041_2025_1251_MOESM4_ESM.tif]
